# A Meta-Analytic Review of the Value of miRNA for Multiple Sclerosis Diagnosis

**DOI:** 10.3389/fneur.2020.00132

**Published:** 2020-02-25

**Authors:** Zongpu Zhou, Huihui Xiong, Fukang Xie, Zhongdao Wu, Ying Feng

**Affiliations:** ^1^Medical School of South China University of Technology, Guangzhou, China; ^2^Histology and Embryology Department of Zhongshan School of Medicine, Sun Yat-sen University, Guangzhou, China; ^3^Parasitology Department of Zhongshan School of Medicine, Sun Yat-sen University, Guangzhou, China; ^4^Key Laboratory of Tropical Disease Control (SYSU), Ministry of Education, Guangzhou, China

**Keywords:** microRNAs, multiple sclerosis, liquid biopsy, serum, meta-analysis

## Abstract

**Backgrounds and Purpose:** Multiple sclerosis (MS) is an immune-mediated chronic inflammatory demyelinating disease of the central nervous system. The etiology of MS is unclear, disease diagnosis mainly based on symptoms, and lacks effective laboratory test index. Circulating microRNAs (miRNAs) as sensitive biomarkers have been widely studied, the expression levels of certain miRNAs are dynamically changed in MS patients. This meta-analysis aims to assess the overall diagnostic accuracy of circulating miRNAs for MS.

**Methods:** We searched PubMed, EMBASE, Cochrane Library, CNKI databases as of July 20, 2019. QUADAS was used to assess the quality of included studies. All studies were processed by Stata 15.0 software. Eleven articles with 600 patients with MS and 389 controls were included.

**Results:** The sensitivity and specificity, PLR, NLR, and DOR of the overall studies were 0.81 (95% CI 0.77–0.84), 0.75 (95% CI 0.68–0.81), 3.3 (95% CI 2.5–4.3), 0.25 (95% CI 0.20–0.32), 13 (95% CI: 8–20), and 0.85 (95% CI 0.82–0.88). Subgroup analysis indicated that miRNA assay had higher diagnostic accuracy for relapsing-remitting MS (RRMS) when compared with other MS subtypes.

**Conclusion:** Our study performed a meta-analysis to generate an estimate of the relevance of miRNA change and the occurrence of MS, and revealed circulating miRNAs has the potential to be used for MS diagnosis, especially for RRMS. Future studies should clarify to which specific miRNAs can accurately diagnose disease subtypes. The miRNA-related pathogenesis may provide theoretical basis for drug development for early intervention.

## Introduction

Multiple sclerosis (MS) is a demyelinating disease of the central nervous system (CNS), which mainly damages cortex, optic nerve, spinal cord, brainstem, and cerebellum. It has the features of spatial and time multiplicity, divided into four types: relapsing-remitting MS (RRMS), secondary progressive MS (SPMS), primary progressive MS (PPMS) and progressive-relapsing MS (PRMS), according to each period's clinical characteristics (1). McDonald Criteria as the golden standard of MS diagnosis is on the basis of clinical symptoms (2), clinical evidence is sufficient for establishment of the diagnosis in most cases. MS patients often initially fall ill with clinically isolated syndrome (CIS) (3), cerebrospinal fluid, magnetic resonance imaging, and electroencephalogram possess auxiliary diagnosis. The prevalence of MS increased in recent year (4). The incidence of MS increase with latitude, northern European countries are representative regions (1). Autoimmune responses play the pivotal effect during the course of MS, in which inflammatory cells and factors damage the myelin sheath surrounding the axon and impair the transmission of nerve impulse (5). CD4^+^ T-cell, particularly T-helper type 1 cells and T-helper type 17 disfunction cause immunological imbalance, along with it inflammatory cytokines level is no longer normal (6), the details behind which have not been totally elucidated yet. Aiming to adjust this out of control state, coadministration of glucocorticoid, and immunosuppressant is conventional therapeutic schedule, plasmapheresis would be applied as necessary (7).

As a new method of early-stage diagnosis, liquid biopsy is attracting more and more attention and expectations, among which small molecular RNA detections are the typical examples. MicroRNAs (miRNA) are non-coding RNAs which only have 20–22 nucleotides, which function as the negative regulator of gene expression at post-transcriptional level in the cell (8). Due to their strong stability, miRNAs can resist RNase A digestion, boiling, and extreme PH condition (9). MicroRNA detection assay has been applied in preclinical stage with the feature of high sensitivity and specificity, especially in cancer researches. High-throughput assay and bioinformatics technology help to seek the potential modulatory miRNAs (10). MiRNAs can be detected in brain circulation due to permeability of blood brain barrier. They had been used as a diagnostic marker for many neurological diseases (11). Combining miRNA assay with other detection technology has become an effective way to improve disease detection rate (12).

Many animal experiments have been carried out on the pathology of demyelinating diseases, such as the famous EAE (Experimental Autoimmune Encephalomyelitis) model (13). It was confirmed that miRNA-155 is essential to CD4^+^ T cells activation, promote the secretion of cytokines by dendritic cells to induce Th17 cell formation in EAE model (14). A category of miRNA is evidenced to participate in repair the central neural system injury and repair. However, the regulation mode of miRNA is complex, presenting network interactions that hardly are explained from one perspective, it is necessary to combine their functions to explain some phenomena (15). For patients with mild symptoms, McDonald Criteria are not diagnostic in some cases. But the earlier the diagnosis and intervention, the better the long-term outcome. The finding of potential diagnostic microRNA is not only valuable for MS but also other neuro-immune disease. Some prospective study detected the expression level from disease progressive phase, followed up with patient to ascertain the predictability miRNA (16), providing a time window for preventive or retardatory treatment in this way. Herein, we analyzed the data from miRNA research of MS patient circulation to infer the correlation between the miRNA and demyelinating diseases.

## Methods

### Search Strategy

All of the publications were searched from PubMed, Web of Science, EMBASE, the Cochrane Library, Chinese National Knowledge Infrastructure (CNKI) databases and related papers up to Jul 20, 2019. Search strategies include: “microRNAs” or “miRNA” or “miR,” and “demyelinating diseases” or “multiple sclerosis” or “clinical isolated syndrome,” and “blood” or “cerebrospinal fluid” or “plasma” or “serum.” The articles in the reference which are related to our subject were also included to avoid choice bias as far as possible. Two investigators independently scrutinized the full text of articles that might qualify.

### Study Selection

The inclusion criteria were as follow: (i) the evaluation experiments must be miRNA assay for multiple sclerosis and the assay is processed at the onset of disease. When a study included both miRNA assay and other tests, we only extracted information on miRNA; (ii) MS patients should certainly be diagnosed by McDonald Criteria; (iii) samples must be collected from patients' circulation and the detection method follow certain strategies, acceptable methods including qPCR, real-time PCR, micro array, and miRNA sequence; (iv) the data in the literature should be sufficient to effectively evaluate the diagnostic performance of circulating miRNA in multiple sclerosis; (v) studies which examined miRNA as a risk factor for other medical conditions, such as taking medicine, were excluded. Letters, reviews, meeting abstracts, and editorials were removed. Publications with repeated and missing data were also excluded.

### Data Extraction and Quality Assessment

The information of included studies was processed severally by two reviewers, and assessed data involved seven aspects: author's first name, published year, country of investigation, sample number, sex ratio of patients, type of miRNA, miRNA detection method, sample sources, disease subtype, specificity, sensitivity, true positives, false positives, false negatives, and true negatives. The summary receiver operating characteristic curves value (SROC) was drown on the basis of sensitivity and specificity, and the area under the curve (AUC) value present a global measurement of test performance. The closer the AUC was to 1, we choose the better values. The Quality Assessment of Diagnostic Accuracy Studies (QUADAS) score system was used to further assess the statistic qualities of included studies (17). The third reviewer did the final estimation, arguments with other reviewers was solved through adjusting certain strategies.

### Statistical Analysis

The raw data were analyzed using STATA 15.0 software. Several common evaluation indicators, including sensitivity, specificity, positive likelihood ratios (PLR), negative likelihood ratios (NLR) diagnostic odds ratio (DOR), and area under the SROC, were used to perform power of miRNA test. The *I*^2^ test was conducted to estimate the proportion of total variation among studies that was due to heterogeneity rather than chance. *I*^2^ value over 50% indicates significant heterogeneity of enrolled studies, and random effects model will be applied in the analysis. The origin of heterogeneity was sought by subgroup and meta-regression analyses, then to assess the influence of these factors to the combined effect size. Quantitative analysis of the publication bias to the included studies was processed by the Deek's test and funnel plots. If there is an asymmetric distribution of data points in the funnel plot, with *P* < 0.05, it illustrates the existence of potential publication bias.

## Results

### Literature Search Process

**Figure 1:**
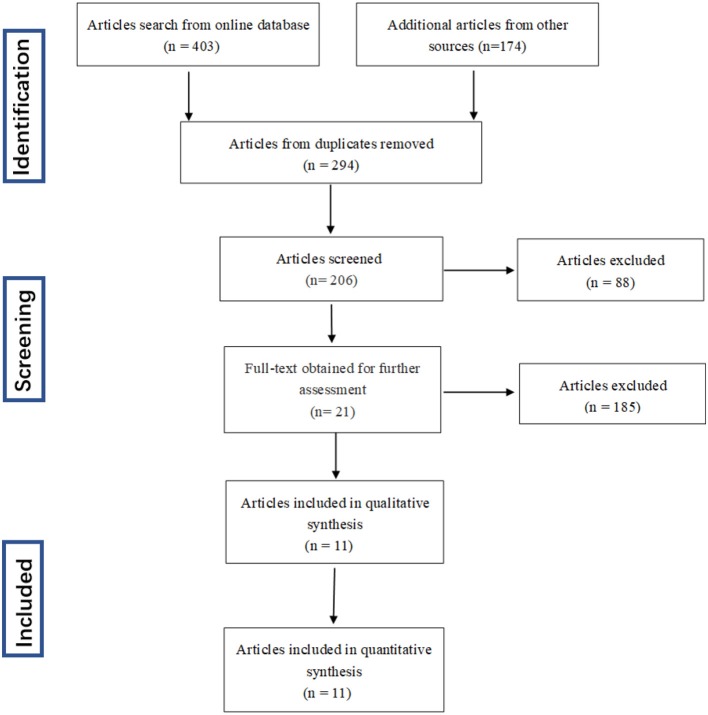
Flow diagram of study selection process.

The process dealing with the searched studies was showed as Figure 1. Firstly, a total of 577 related papers were retrieved from the relevant databases by the search method above mentioned. Through reading titles and abstracts, 88 articles were excluded. Remaining citations conform to our subject, but after reading the full text, we were incapable to construct a 4-fold table for 185 papers, 20 papers included patient's data which is disturbed by medication, 1 study did not clearly describe the diagnostic criteria. After excluding letters, reviews, and meta-analysis, three articles were added through retrospective research after reading the reference publications. Eleven articles were finally enrolled in this meta-analysis (16, 18–27).

### Basic Characteristics and Quality Assessment of Included Studies

Basic characteristics and quality assessment of included studies were presented in Table 1. Eleven articles with 600 patients with MS and 389 controls were included in total. The enrolled articles were published before 2019. The ethnicity of all patients are Caucasians. The 11 eligible articles contain half single miRNA assays and half multiple miRNA assay. Further sample of seven studies was collected from serum, while the others were collected from blood, plasma or cerebrospinal fluid. The technology of detecting target genes in those studies was based on reverse transcription polymerase chain reaction (RT-PCR) or micro-assay. The result for quality of enrolled studies which was assessed by QUADAS, all of them are relatively high.

**Table 1:** Basic information of included studies.

**References**	**Country**	**Research method**	**Case number**	**Female ratio**	**Control number**	**Female ratio**	**Studied microRNA**	**Detection technique**	**Specimen source**
Ebrahimkhan et al. (22)	Italy	Prospective trial	25	0.60	11	0.82	miR-15b-5p, miR-451a, miR-30b-5p, miR-342-3p; miR-127-3p, miR-370-3p, miR-409-3p, miR-432-5p	Small RNA sequencing	Serum
Sharaf-Eldin et al. (23)	Egypt	Prospective trial	37	0.76	23	0.74	miR-145 and miR-223	TaqMan MicroRNA assays	Serum
Selmaj et al. (24)	Poland	Prospective trial	33	0.76	32	0.75	miR-122-5p, miR-196b-5p, miR-301a-3p, miR-532-5p	Digital quantitative PCR	Serum
Regev et al. (25)	America	Prospective trial	48	0.71	30	0.83	miR-484, miR-140-5p, miR-320a, miR-486-5p, miR-320c	LNA SYBR green–based real-time PCR	Serum
Keller et al. (20)	Germany	Prospective trial	50	0.72	50	0.72	miR-7-1-3p,miR-7-1-3p	Next-generation sequencing	Blood
Vistbakka et al. (19)	Finland	Prospective trial	62	0.68	21	0.57	miR-191-5p	RT-PCR	Serum
Sondergaard et al. (21)	Denmark	Prospective trial	22	0.64	15	0.67	mi-RNA-145	Locked nucleic acid-based mi-RCURY microarray	Plasma
			40	0.63	40	0.53	mi-RNA-145		Serum
Mancus et al. (26)	Italy	Prospective trial	62	0.58	15	0.87	miR-572	Real time PCR system	Serum
Gandhi et al. (18)	America	Prospective trial	10	0.80	9	0.56	miR-30e	real-time PCR (RT-PCR)	Blood
Bergma et al. (27)	Sweden	Prospective trial	181	n.a	115	n.a	miR-150	TaqMan microRNA Reverse Transcription Kit	CSF
Ahlbrec et al. (16)	Germany	Prospective trial	30	0.77	28	0.75	miRNA-181c	TaqMan microRNA reverse transcription kit	CSF

### Diagnostic Accuracy of Circulating miRNA in MS

**Figure 2:**
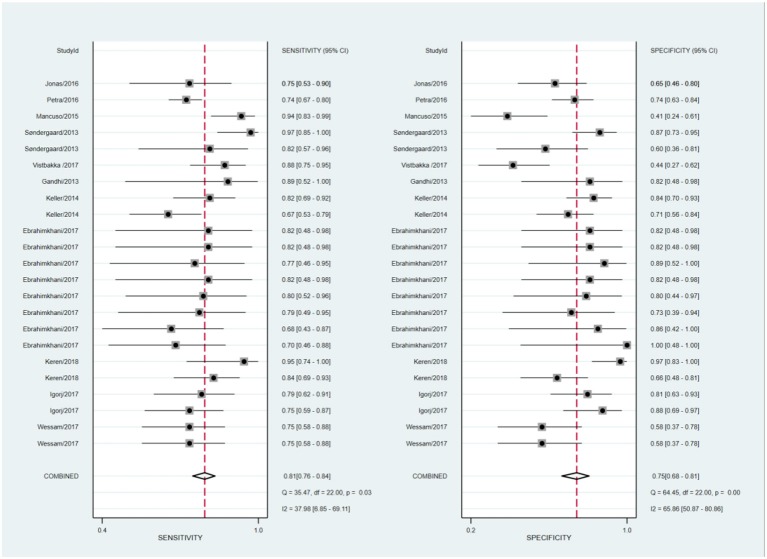
Forest plots of sensitivity and specificity with corresponding heterogeneity statistics.

**Figure 3:**
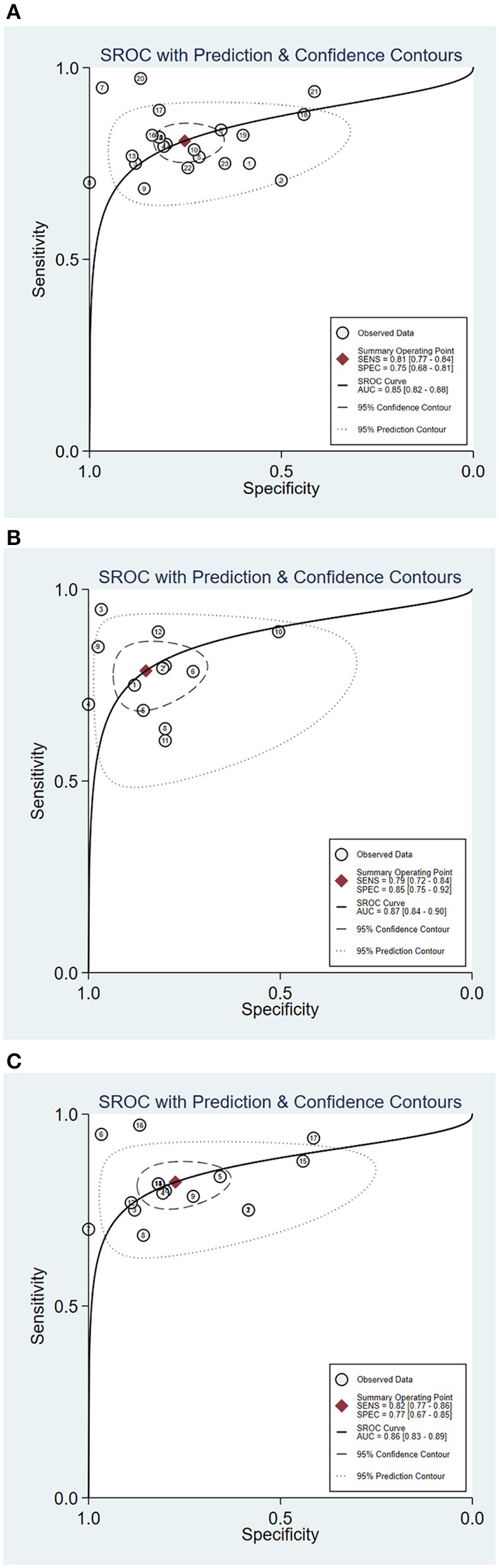
Summary receiver operator characteristic (SROC) curve of circulating miRNA assay for MS [**(A)** SROC of overall studies; **(B)** SROC for RRMS patient; **(C)** SROC of serum miRNA-based studies].

The statistical approach of meta-analysis is generally divided into two steps. Firstly, heterogeneity test was processed to assess the consistency of research results and chose effects model. The *I*^2^ test of overall heterogeneity for specificity were 67.65%, which reminded the heterogeneity among studies was evident (*I*^2^ > 50%), hence the random effect model was applied (Figure 2). The second step was to combine the effect size of each study. The sensitivity and specificity, PLR, NLR, and DOR of the selected studies were 0.81 (95% confidence interval (CI) 0.77–0.84), 0.75 (95% CI 0.68–0.81), 3.3 (95% CI 2.5–4.3), 0.25 (95% CI 0.20–0.32), 13 (95% CI 8–20), and 0.85 (95% CI 0.82–0.88), respectively (Figure 3A). We also drew Fagan's plot, includes pre-test probability and posttest probabilities, it described the change to the MS diagnosis of miRNA assays. Any subject had the same pre-test probability to suffer MS, which was 20%, if the result of miRNA test was positive, PLR value was 3, and the posttest probability with MS rose to 45%; similarly, the negative result of miRNA test would lower the posttest probability to 6%. Therefore, miRNA test had certain potential to improve the diagnostic efficiency of MS.

### Subgroup Analyses and Meta-Regression

**Figure 4:**
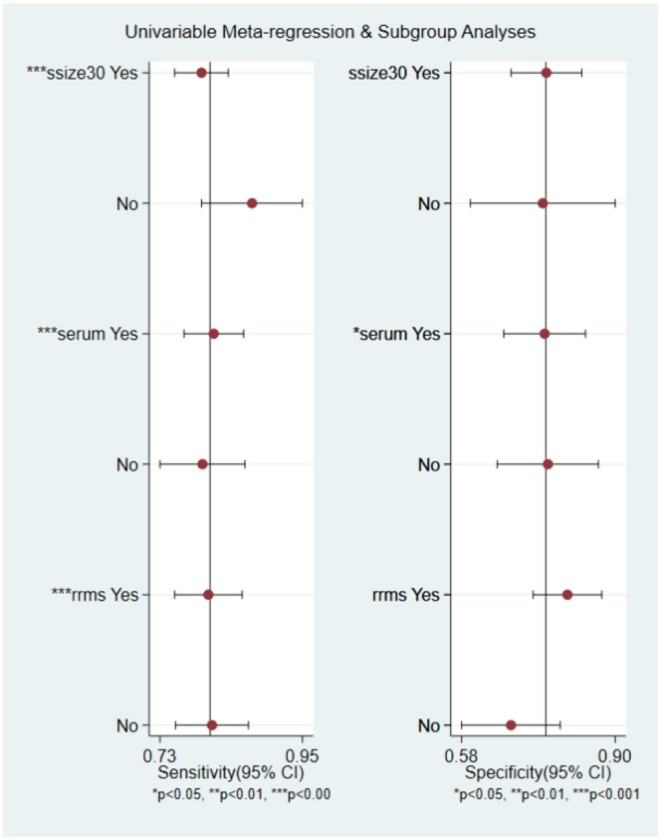
Univariable meta-regression analysis of study design parameters on the for source of heterogeneity in sensitivity and specificity.

In order to explore the potential sources of heterogeneity among included studies, subgroup and multivariate meta regression analyses were further performed. As shown in Figure 3B, subgroup analysis based on miRNA profile suggested that miRNA assay for RRMS showed a better diagnosis performance than others types. MiRNA test for RRMS revealed a high specificity of 0.87(95% CI 0.75–0.92), PLR value was 5.3 (95% CI 3.1–9.1), NLR value was 0.25 (95% CI 0.19–0.34), DOR was 21 (95% CI 10–42), and AUC was 0.87 (95% CI 0.84–0.90), respectively. Additionally, the source of sample influences the quality of detection, the assay for patient's serum (Figure 3C) showed a relatively high diagnostic accuracy and the corresponding results sensitivity, specificity, PLR, NLR, DOR, and AUC were 0.82 (95% CI 0.77–0.86), 0.77 (95% CI 0.67–0.85), 3.6 (95% CI 2.5–5.4), 0.23 (95% CI 0.18–0.30), 16 (95% CI 9–28), 0.86 (95% CI 0.83–0.89), respectively. Univariate meta-regression analyses were then processed to identify whether the inter-study heterogeneity sourced from sample size, sources of controls and subjects, quality of reference test, miRNA profile (Figure 4). Overall, the primary source for heterogeneity of sensitivity was the difference in miRNA sources with an extremely significant (*P* < 0.001), combining aforementioned results, which may present that serum miRNA assay for RRMS diagnosis had better diagnostic efficiency. In addition, changing the number of samples only cause any significant heterogeneity for sensitivity but not specificity, indicating higher sample size is beneficial to the potential discovery of new markers.

### Robustness Analysis and Publication Bias

**Figure 5:**
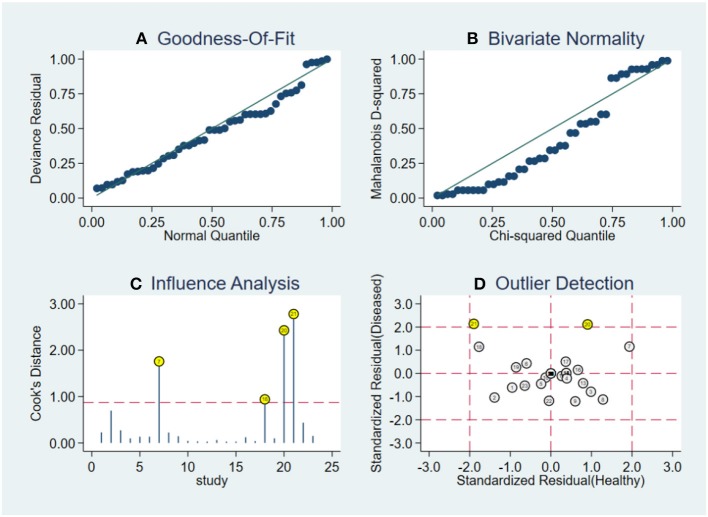
Robustness analysis, influence analysis, and outlier detection. **(A)** Goodness of fit, **(B)** bivariate normality, **(C)** influence analysis, and **(D)** outlier detection.

**Figure 6:**
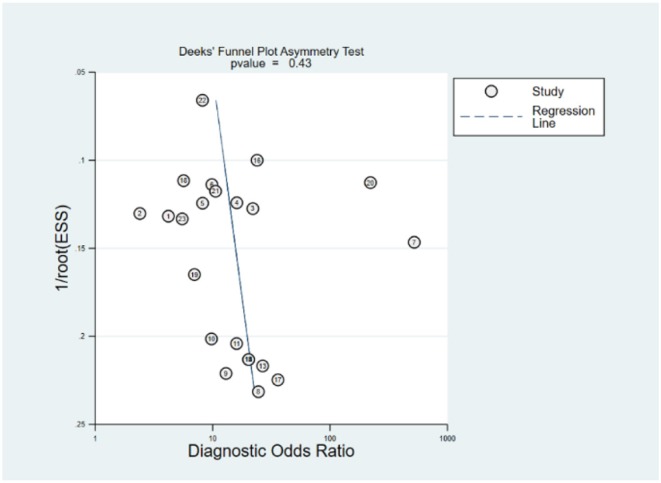
Funnel plot for publication bias of circulating miRNA assay.

Robustness analysis was processed to assess the result reliability (Figure 5). The model involved in the statistical analysis was valid and robust, and had been verified by goodness of fit and binary normality analysis. Influence analysis and outlier detection identified two outlier studies. The overall results did not reveal any significant changes after these outliers were excluded (Table 2). At last, we used Deeks' funnel plot asymmetry test to investigate the publication bias influence. The *P*-value of publication bias for overall miRNA assay was 0.43 (Figure 6), which was a non-significant value and indicated little possibility of publication bias.

**Table 2:** Summary estimates of diagnostic performance of miRNAs in NDs detection.

**Analysis**	**SEN (95% CI)**	**SPE (95% CI)**	**PLR (95% CI)**	**NLR (95% CI)**	**DOR (95% CI)**	**AUC (95% CI)**
**Disease type**						
RRMS	0.79 (0.72–0.84)	0.85 (0.75–0.92)	5.3 (3.1–9.1)	0.25 (0.19–0.34)	21 (10–42)	0.87 (0.84–0.90)
SPMS or PPMS	0.80 (0.70–0.87)	0.76 (0.69–0.83)	3.4 (2.4–4.9)	0.26 (0.16–0.43)	13 (6–30)	0.84 (0.81–0.87)
**Sample source**						
Serum	0.82 (0.77–0.86)	0.77 (0.67–0.85)	3.6 (2.5–5.4)	0.23 (0.18–0.30)	16 (9–28)	0.86 (0.83–0.89)
Other sources	0.80 (0.73–0.86)	0.68 (0.57–0.78)	2.5 (1.8–3.5)	0.29 (0.22–0.39)	9 (5–14)	0.82 (0.79–0.85)
**Mi-RNA profile**						
Single mi-RNA	0.87 (0.77–0.92)	0.66 (0.52–0.78)	2.5 (1.7–3.8)	0.20 (0.11–0.36)	13 (5–29)	0.85 (0.82–0.88)
Multiple mi-RNA	0.78 (0.73–0.82)	0.79 (0.72–0.85)	3.7 (2.7–5.1)	0.28 (0.23–0.35)	13 (8–22)	0.82 (0.79–0.86)
Overall	0.81 (0.76–0.84)	0.75 (0.68–0.81)	3.3 (2.5–4.3)	0.26 (0.21–0.32)	13 (8–19)	0.85 (0.82–0.88)
Outlier excluded	0.77 (0.73–0.81)	0.76 (0.69–0.81)	3.2 (2.4–4.1)	0.30 (0.25–0.36)	11 (7–16)	0.80 (0.76–0.83)

*CI, confidence interval; SEN, sensitivity; SPE, specificity; PLR, positive likelihood ratio; NLR, negative likelihood ratio; DOR, diagnostic odds ratio; AUC, area under the curve*.

## Discussion

Multiple sclerosis is a chronic inflammatory demyelination disease with impaired CNS function, disease symptoms are heterogeneous and mainly associated with pyramidal tract damage, once the disease enters progressive-relapsing phase, patients manifested as asymmetric paralysis, paresthesia, and ataxia (1). Young people, especially women, account for the most portion of the affected population (28, 29). Early and accurate diagnosis of MS could greatly reduce the burden on society caused by this chronic disease. In our study, we performed a systematic review of studies related to circulating miRNAs expression in MS. The diagnostic ability of miRNAs in previous studies remains controversial. Significant miRNA expression variations have been detected in MS patients and related animal models, mainly in inflammatory cells (30, 31), the predictive effect of each miRNA for MS is different. In particular, miR-125a-5p in the blood distinguishes MS patients from healthy controls with high specificity of 85% but low sensitivity of 56%, inversely, miR-25a-3p has sensitivity of 75% but low specificity of 58% (32). Therefore, we intended to comprehensively assess the diagnostic accuracy of circulating miRNAs for MS by including a large-size data sample. We collected the candidate circulating miRNAs from 11 articles which applied McDonald Criteria as the gold standards. We found the good diagnostic performance for MS by miRNA assays, with pooled sensitivity and specificity 0.81 (95% CI 0.77–0.84), 0.75 (95% CI 0.68–0.81), respectively, and the AUC is 0.85 (95% CI 0.82–0.88). However, part of miRNAs expression elevates while the others decline, and it must be noted that heterogeneity existed in the included studies, which was mainly ascribed to the types of miRNAs sample. Subgroup analyses suggested that miRNAs in RRMS patients has moderately better diagnostic efficiency than other types, specificity up to 0.87. Our results are likely to be generalizable to studies of other diseases beyond multiple sclerosis, because most biases described herein are not specific to multiple sclerosis but other peripheral demyelinating diseases as well, such as Guillain-Barré syndrome mediated by immune inflammatory response, may have miRNA diagnostic value similar to MS, and can be further studied. Particularly, it is the first meta-analysis centering on miRNAs in MS, with systemically quantitative evaluation for the diagnostic value.

MiRNAs as a post-transcriptional regulator control mRNA translational inhibition or degradation. Through packing miRNA in exosomes to induce the target cell gene expression change enable intercellular interactions. MiRNAs account for the highest proportion of small non-coding RNAs (33). They are often regarded as biomarkers for its strong stability and detectability by RT-PCR technique. In recent year, insights into the role of miRNAs in cancer make miRNAs an attractive tool for disease screening (10). Function studies have suggested miRNAs target to gene of immuno-inflammatory responses, the validation of these post-transcriptional regulation has enabled a better understanding of MS pathogenesis at the molecule level. MiRNAs are found throughout the body, in the original lesions, plasma, serum, interstitial fluid (34). The conclusion mentioned above suggest that serum miRNAs served as biomarkers had higher diagnostic accuracy than other sources. However, in a previous study about neurodegenerative diseases, blood and plasma samples provide more valuable information for diagnosis, and miRNAs concentration in plasma is higher than serum (35). There are many other researches, such as one related to leukemia, which is similar with us, reminds serum results are more meaningful (36). As we all know ribonuclease exist in serum, the miRNA that can be detected must be resistant to the digestion of ribonucleases. And the serum miRNAs result would be contaminated by miRNAs released from cells, either from hemolysis, or remaining whole cell during sample processing (37). Moreover, it is inevitable that the serum sample will be affected by other small RNAs and broken nucleic acid fragments (9). In order to eliminate these factors in further studies, it is very important to properly set up normal control, optimized measurement technique and apply suitable statistical analysis. Redundancy effect may lead to a one-sided conclusion in miRNA research (38), that is, if one miRNA loses its function, alternate miRNA may offset the deficiency by other pathway. In addition, the lesions of MS are mainly concentrated in the CNS. It has been widely perceived that cerebrospinal fluid (CSF) sample result should be more suggestive. But most studies have proved that is not true, which further indicates that MS is the result of systemic autoimmune response.

There is a vital issue surrounding miRNA research, that is the comparison of single and multiple miRNA assay. Single miRNA assay has relatively higher sensitivity and AUC value in our study. MiRNA-155 mentioned above is a typical example, which is associated with activation of inflammatory cells, damage of the blood-brain barrier, and neurodegenerative processes. Significant change of miRNA-155 have been detected in samples of MS patients, and similar findings exist in animal models (39). Relatively speaking, multiple miRNA detection methods are more comprehensive, but at the same time the results receive more impacts, so the analysis of the results should be more prudent. Combining the data characteristics of the included studies and whole population incidence study, MS is sex dependent and mainly affects women, hormone levels affecting immune system response had been reported (28). Even in female group, miRNA expression pattern differs from one to another. One study about sncRNA (microRNA & snoRNA) detected that the expression change of 38 sncRNAs only happen in females, and a set of uniform sncRNAs alteration in the remission phase of MS were mainly detected in samples from female patients (40). During the sample collection process, samples without pharmacological intervention tend to be integrated by researchers, and the same is true in our study. Most patients take medicine after having a definite diagnosis, these patients' data are usually excluded. On the other hand, miRNAs would be used as therapeutic response biomarkers for MS patients who have taken medicine. Classic drugs treated MS include interferon-β, glatiramer acetate, natalizumab, and fingolimod (41). Fingolimod as star drug for RRMS had been discovered to modulate miR-15b, miR23a and miR-223, they show varying expression levels at each stages of the disease (42), prognostic indicators can be formulated through connecting known miRNA mechanism, which can also provide evidence for the effectiveness of the treatment regimen.

Although our research is innovative, there are still many limitations. Firstly, the number of studies included is limited. Expanding the sample size makes the conclusion more exhaustive and convincing. Subgroup analysis can provide more clinical guidance. Secondly, SPMS, PPMS, and PRMS should be studied as subtypes, just like RRMS. Many of the included studies did not provide information on the type of disease, and although the incidence of RRMS was the highest, not every MS patient was diagnosed with RRMS at the outset. Thirdly, the reference miRNAs were inconsistent in the inclusion studies, and different research teams were accustomed to different references, which are not mentioned in some articles. Although the detection methods they use are based on RT-PCR, each method of detection is not identical. The above factors may lead to different conclusions.

This meta-analysis suggests that miRNAs have reference value for MS diagnosis. Subgroup analysis indicate serum or single miRNA assay has better diagnostic accuracy, and the assay is more effective for RRMS diagnosis. Our article merely summarizes the overall effect of miRNAs, each subtype still requires their own specific miRNAs. There is some heterogeneity in our study, large prospective cohort study is needed to further prove the significance of miRNA for MS.

## Data Availability Statement

The raw data supporting the conclusions of this article will be made available by the authors, without undue reservation, to any qualified researcher.

## Author Contributions

YF conceived the study and wrote the paper. ZZ and HX performed the literature search, data extraction, and analyzed and interpreted data. FX and ZW revised the manuscript.

### Conflict of Interest

The authors declare that the research was conducted in the absence of any commercial or financial relationships that could be construed as a potential conflict of interest.
